# The Development of Indonesian Online Game Addiction Questionnaire

**DOI:** 10.1371/journal.pone.0061098

**Published:** 2013-04-03

**Authors:** Tjibeng Jap, Sri Tiatri, Edo Sebastian Jaya, Mekar Sari Suteja

**Affiliations:** 1 Science, Technology, and Society (STS) Research Group, Tarumanagara University, Jakarta, Indonesia; 2 Faculty of Information Technology, Tarumanagara University, Jakarta, Indonesia; 3 Faculty of Psychology, Tarumanagara University, Jakarta, Indonesia; The University of Queensland, Australia

## Abstract

Online game is an increasingly popular source of entertainment for all ages, with relatively prevalent negative consequences. Addiction is a problem that has received much attention. This research aims to develop a measure of online game addiction for Indonesian children and adolescents. The Indonesian Online Game Addiction Questionnaire draws from earlier theories and research on the internet and game addiction. Its construction is further enriched by including findings from qualitative interviews and field observation to ensure appropriate expression of the items. The measure consists of 7 items with a 5-point Likert Scale. It is validated by testing 1,477 Indonesian junior and senior high school students from several schools in Manado, Medan, Pontianak, and Yogyakarta. The validation evidence is shown by item-total correlation and criterion validity. The Indonesian Online Game Addiction Questionnaire has good item-total correlation (ranging from 0.29 to 0.55) and acceptable reliability (α = 0.73). It is also moderately correlated with the participant's longest time record to play online games (r = 0.39; p<0.01), average days per week in playing online games (ρ = 0.43; p<0.01), average hours per days in playing online games (ρ = 0.41; p<0.01), and monthly expenditure for online games (ρ = 0.30; p<0.01). Furthermore, we created a clinical cut-off estimate by combining criteria and population norm. The clinical cut-off estimate showed that the score of 14 to 21 may indicate mild online game addiction, and the score of 22 and above may indicate online game addiction. Overall, the result shows that Indonesian Online Game Addiction Questionnaire has sufficient psychometric property for research use, as well as limited clinical application.

## Introduction

Online games has swiftly become a popular source of entertainment for all ages, particularly among young people. It is becoming a regular source of entertainment globally, spreading in conjunction with the constant improvement of internet access. The same phenomenon also occurs in Indonesia, following the improvement of internet access. Online games can be played in the computer, handheld devices, or video game consoles.

The popularity of online games in Indonesia can be easily recognised with the events that are made by game companies or internet cafes. Even a popular institution like *Museum Rekor Dunia Indonesia* (MURI), which holds unique records of activities or certain achievements in Indonesia, hosts an event involving hundreds of participants to break the previous 150 hours record for playing games, most of the games are online games[1]. In Indonesia, most online game players play with friends in internet cafes. The popularity of internet cafes as a place for playing online games is prevalent in both urban areas like Jakarta[2] and rural areas like Pesawaran, Lampung[3].

Besides being a source of entertainment, online games also have potential problems. News regarding the downside of online games should not be unfamiliar anymore. There have been news reported in Indonesia on children and adolescents stealing due to lack of money for playing games in internet cafes[4], [5]. Problems from the use of online games has caused the Indonesian minister of health to publicly express her concern regarding the popularity of online games among children[6].

There have been many problems documented that is linked to playing online games, such as: aggression[7], physical injury[8], and addiction[8], [9]. But among all problems related to online game use, addiction is arguably the most worrying. Online game addiction has been recognised internationally and steps have been taken. South Korea recognised that internet addiction is a nationwide problem and has trained counsellors to be placed in hospitals and treatment centres[10]. Furthermore, preventive measures introduced in schools have also been taken[11]. Similarly, China has recognised the danger and has passed laws that discourage more than 3 hours of daily game use[12]. America has also recognised the problem and prepare for a diagnostic guideline in the upcoming DSM-V[13]. Meanwhile, Indonesia does not have a diagnostic guideline to face the rising online game problems.

The excessive use of online games has been termed differently according to their different theoretical backgrounds. Some of the terms used to describe the problem are: addiction[13], pathological gaming[14], and videogame dependence[15]. However, the term addiction has been commonly used by researchers[8], [9], [13] and is familiar with the Indonesian lay population[4], [5]. Furthermore, behavioral addiction, including online game addiction, has many similarities (chronic, relapse, withdrawal, tolerance, genetic contribution, neurobiological mechanism, response to treatment) with substance addiction[16].

The most frequently used diagnostic criteria for online game diagnosis is an adapted version of Pathological Gambling diagnostic criteria from DSM-IV-TR[17]. The same Pathological Gambling diagnostic criteria are also adapted for Young's Internet Addiction diagnostic criteria[18]. Another set of diagnostic criteria that can be used for online game addiction is proposed by Griffith, which includes salience, mood modification, tolerance, withdrawal, conflict, and relapse[19]. Both, Young's[20] and Griffith's[21] diagnostic criteria have been translated to an online game addiction measure.

This research aims to develop an online game addiction measure to screen for online game addiction cases among Indonesian children and adolescents, as well as for other research purposes. The measure will adopt Pathological Gambling diagnostic criteria from DSM-IV-TR[17] and Griffith's addiction criteria[19]. We are aware that online game addiction measures have been previously constructed [20]–[22]. But, there have been no previous research that attempt to construct an online game addiction measure that is specific to the context of Indonesian children and adolescents. Cultural context is important to consider as different cultures would have different expressions of psychological problems [23], [24]. In the development of Indonesian online game addiction questionnaire, we pay close attention to the expression of online game addiction among Indonesian children and adolescents by field observations and discussions. Then, the newly developed measure's psychometric properties will be examined. We will also attempt to construct a cut-off score to provide an estimate of mild addiction and addiction category.

## Method

### Ethics Statement

This study was approved by the Human Research Ethics Committee of Institute for Research and Academic Publications, Tarumanagara University, Indonesia. Written informed consent was obtained from the school's principal. The school's principal act as the guardians and caretaker of the children participants involved in the study. The school's principal is responsible for the guardians and caretaker of the children in school, where we conducted our research.

### Participants

The research participants are school students from several schools in Manado, Medan, Pontianak, and Yogyakarta. The cities are chosen to represent the wide Indonesia archipelago; Manado is chosen to represent Celebes islands, Yogyakarta is chosen to represent Java, Medan to represent Sumatera, and Pontianak to represent Borneo. The number of private and public schools chosen is even.

The initial participants are 3,264 junior and senior high school students. However, only 45.3% of them played online games at least once in the last month and have not decided to stop, which amounts to 1,477 participants. There are 907 junior high school students and 570 senior high school students. The gender is quite even between male (N = 882) and female (N = 590). Most of the participants have a weekly allowance between Rp 20.000–Rp 50.000 (30.5%, N = 449), followed by below Rp 20.000 (22.7%, N = 334), Rp 50.000–Rp 100.000 (22.4%, N = 330), and Rp 100.000–Rp 200.000 (10.8%, N = 159). The mean age of the participants is 14.21 years old (SD = 1.78).

### Validation

The validation will use internal consistency to indicate construct homogeneity. Furthermore, the measure will be correlated with several objective self-report questions for criterion validity. The self-report questions are: longest time recorded in playing online games, average days per week in playing online games, average hours per days in playing online games, weekly expenditure for internet cafes, and monthly expenditure for online games. All of them can be an indicator of excessive use of online games that can be related to addiction. On the other hand, the measure will also be correlated with further self-report questions that may produce negative correlation (time spent to do school work).

### Measure construction

The Indonesian Online Game Addiction Questionnaire is developed from previous findings, as well as qualitative work to capture the Indonesian expression of online game addiction. This measure is particularly informed by previous similar game addiction measurement development [21]–[23]. But, the Indonesian online game addiction questionnaire is based on both, Pathological Gambling criteria from DSM-IV-TR[17] and Griffith's addiction criteria[19]. Before writing the items, we conducted interviews and focus group discussions to junior and senior high school students to gather information regarding the characteristic of excessive online games use. We also conduct field observations to internet cafes that are popular among school students and interviewed several players that are in school uniform. The qualitative work is important to make sure that the items written will be natural to Indonesian school students living condition.

Then, we wrote items that are based on diagnostic guidelines and qualitative data. The first item is on thinking about online game for the whole day, reflecting preoccupation[17] or salience[19]. The second is about increasing use of online game, which reflects the second criterion of Pathological Gambling[17] or tolerance[19]. The third item emphasizes the use of online game as a media to escape real life, it reflects the fifth criterion of Pathological Gambling[17] or mood modification[19]. The fourth item is about relapse, but it is quite different from the diagnostic guideline. Similar to the third Pathological Gambling criterion, but this item underlines the role of others in stopping the addiction. The fifth item is about the withdrawal effect of not playing online games, drawn from the fourth criterion of Pathological Gambling[17] or withdrawal[19]. The sixth item is on the conflict caused by playing online games, drawn from the ninth criterion of Pathological Gambling[17] or conflict[19]. However, the items are written with moderation in context to Indonesian school students. The original criterion of Pathological Gambling used the terms ‘jeopardized’ or ‘loss’ a significant relationship. The term is not very appropriate to Indonesian context as it would make them defensive. As relationship is of the utmost importance in a collective culture, like Indonesia, using such a harsh term would put participants in the defensive. Therefore, we decide to moderate the term as ‘problems with family or friends’. Finally, the seventh item is about sleep deprivation. The item is aimed to tap on the problem that often arises due to excessive online game use. Qualitative data showed that sleep deprivation is the strongest indicator of excessive online game use. In addition, school students identified sleep deprivation as one of the main problem of excessive online game use.

Furthermore, the measure is to be answered according to the condition of the last six months. The items are to be answered on 5 points Likert Scale, ranging from Never to Very Often. The participants are instructed to think about the frequency they did the activities described on the items, according to the last six months.

## Result

### Descriptive result

The descriptive results of the validation sample are shown in Table 1. The mean of the online game addiction scale for the online gamers is 14.23 (4.81). The mean for the longest time recorded in playingonline game is 5.79 hours (5.12), with a maximum score of 82 hours.

**Table 1:** Descriptive results of the online game behaviour on the validation sample.

Measures	Result
Indonesian Online Game Addiction Questionnaire	M = 14.23 (SD = 4.81)
Longest time record to play online games	M = 5.79 hours (SD = 5.12)
Average days per week in playing online games:	
1 day/week	19.10% (N = 281)
2–3 days/week	37.40% (N = 552)
4–5 days/week	14.20% (N = 210)
6–7 days/week	14.20% (N = 210)
Missing	15.20% (N = 224)
Average hours per days in playing online games:	
0–½ hours	6.6% (N = 97)
>½–1 hours	8.4% (N = 124)
>1–2 hours	21.7% (N = 320)
>2–3 hours	21.1% (N = 312)
>3–4 hours	11.7% (N = 173)
>4–5 hours	8.7% (N = 128)
>5–6 hours	4.5% (N = 67)
>6–7 hours	1.7% (N = 25)
>7 hours	3.9% (N = 58)
Missing	11.8% (N = 153)
Weekly expenditure for internet cafes:	
None	57.3% (N = 847)
Below Rp 20.000,-	26.3% (N = 388)
Rp. 20.000,- to Rp. 50.000,-	10.0% (N = 148)
Rp. 50.000,- to Rp. 100.000,-	3.5% (N = 52)
Rp. 100.000,- to Rp. 200.000,-	1.4% (N = 21)
Rp. 200.000,- to Rp. 300.000,-	0.4% (N = 6)
Above Rp. 300.000,-	0.1% (N = 2)
Missing	0.9% (N = 1)
Monthly expenditure for online games:	
None	42.2% (N = 623)
Below Rp 20.000,-	15.0% (N = 222)
Rp. 20.000,- to Rp. 50.000,-	17.7% (N = 261)
Rp. 50.000,- to Rp. 100.000,-	9.6% (N = 142)
Rp. 100.000,- to Rp. 200.000,-	7.8% (N = 115)
Rp. 200.000,- to Rp. 300.000,-	3.4% (N = 50)
Above Rp. 300.000,-	1.6% (N = 23)
Missing	2.8% (N = 41)

### Validity analysis

The Indonesian Online Game Addiction Questionnaire will be examined for its construct homogeneity using Cronbach Alpha and its criterion validity using correlation with several criteria. The reliability of the scale is α = 0.73. This shows that the scale have acceptable reliability. Furthermore, the item-total correlation ranged from 0.29 to 0.55 which indicated acceptable construct homogeneity. The item-total correlation result is shown in Table 2.

**Table 2:** Item-total correlation of Indonesian Online Game Addiction Questionnaire.

Items	Means	SD	Item-total correlation
*Saya memikirkan game online sepanjang hari.*(I think about playing online game all day long.)	2.29	1.06	0.49
*Waktu bermain game online saya bertambah (misalnya dari 1 jam menjadi 2 jam setiap kali main).*(My online game play time increases (for example: from 1 hour for each game time to 2 hours))	2.31	1.04	0.47
*Saya bermain game online untuk melarikan diri dari masalah.*(I play online game to run away from problems)	2.09	1.20	0.38
*Orang lain gagal saat mencoba untuk membantu saya mengurangi waktu bermain game online.*(Others fail when they try to reduce my online game time)	1.97	1.08	0.52
*Saya merasa tidak enak ketika tidak dapat bermain game online.*(I feel uncomfortable if I cannot play online game)	2.13	1.17	0.55
*Bermain game online membuat hubungan saya dengan orang lain (keluarga, teman, dll) menjadi bermasalah.*(Online game made my relationship with others (families, friends, etc.) problematic)	1.47	0.87	0.29
*Waktu yang saya habiskan untuk bermain game online membuat saya kekurangan jam tidur.*(The time spent to play online games made me lose sleeping time)	2.05	1.24	0.42

The correlation for the criterion validity used Pearson correlation for the interval data (longest time record to play online games) and Spearman correlation for the ordinal data. The Indonesian Online Game Addiction Questionnaire have moderate correlation with longest time record to play online games (r = 0.39; p<0.01), average days per week in playing online games (ρ = 0.43; p<0.01), average hours per days in playing online games (ρ = 0.41; p<0.01), and monthly expenditure for online games (ρ = 0.30; p<0.01). In addition, it is weakly correlated with weekly expenditure for internet cafes (ρ = 0.25; p<0.01). The Indonesian Online Game Addiction Questionnaire also has negative weak correlation with the time spent to do school work (ρ = −0.10; p<0.01). The criterion validity result is shown in Table 3.

**Table 3:** Criterion validity of Indonesian Online Game Addiction Questionnaire.

Criterion	Correlation	Strength
Longest time record to play online games	*r* = 0.39; p<0.01	Moderate
Average days per week in playing online games	ρ = 0.43; p<0.01	Moderate
Average hours per days in playing online games	ρ = 0.41; p<0.01	Moderate
Weekly expenditure for internet cafes	ρ = 0.25; p<0.01	Weak
Monthly expenditure for online games	ρ = 0.30; p<0.01	Moderate
Time spent to do school work	ρ = −0.10; p<0.01	Weak

### Clinical cut-off construction

To construct a clinical cut-off estimate, we will use a combination of criterion and population norm. Three licensed clinical psychologists did a focus group discussion and agreed that regularly playing online games for 4–5 days a week and, on average, spending more than 4 hours each day on online games may be an indication of addiction. The decision regarding the hours is informed by the laws passed in China that discourage more than 3 hours of game use per day[12], as well as the prevailing 3 hours internet café promotion package drawn from field observation. Furthermore, decision on the days per week is based on the idea of a healthy relationship of work and play. We argue that playing online games for more than 4 hours at the frequency of working days is already half of the amount of the standard daily 8 hours working time, thus it can be considered as excessive.

Based on the criteria, a clinical sample is drawn from the original validation sample. There are 150 participants (10.15%) that met the criteria from the total of 1,477 participants. This sample is used to establish the cut-off score.

The clinical sample has similar characteristics, as well as differences from the original validation sample. The similarities are their weekly allowance, monthly expenditure for online games, and weekly expenditure for internet cafes. The majority have between Rp 20.000–Rp 50.000 (28.7%, N = 43) for weekly allowance. They also have no spending for monthly expenditure for online games (26.7%, N = 40) and weekly expenditure for internet cafes (46.7%, N = 70). The distinct differences are the participants' gender and longest time record to play online games. The clinical sample is mainly composed of males participants (81.3%, N = 122). In addition, the longest time record to play online games is averaged at 11.02 hours (SD = 7.47).

Then, we conducted a hierarchical cluster analysis to establish the cut-off score for mild addiction and addiction. The hierarchical cluster analysis done using Ward's method produced two clusters. An ANOVA on the two clusters showed that they have statistically significant difference (F (1, 147) = 247.66, p<0.01). The first cluster is named addiction (N = 89), with the mean of 22.38 (SD = 3.38) and the 95% Confidence Interval for Mean of 21.67 to 23.09. The second cluster is classified as mild addiction (N = 60), with the mean of 14.51 (SD = 2.30) and the 95% Confidence Interval for Mean of 13.92 to 15.11. Therefore, the score of 14 to 21 may indicate mild online game addiction and the score of 22 and above may indicate online game addiction.

## Discussion

The result showed that the Indonesian Online Game Addiction Questionnaire have adequate psychometric properties. The measure is moderately correlated to indicators of excessive online game use, such as: longest time recorded in playing online games, average days per week in playing online games, average hours per days in playing online games, and monthly expenditure for online games. It is also negatively correlated to the time spent to do school work. Additionally, the scale have good item-total score correlation and acceptable reliability.

Currently, the Indonesian Online Game Addiction Questionnaire is best used for research purposes. However, it must be noted that this measure is constructed and validated on the context of Indonesian school students. Therefore, it is best used for research with Indonesian school students. The measure might not yield optimal results if it is used outside the context of Indonesian school students (i.e. adult population).

Although limited, the Indonesian Online Game Addiction Questionnaire may also be used for clinical purposes. It may provide an estimate of online game addiction prevalent in a population. The Indonesian Online Game Addiction Questionnaire can also be used as a supporting evidence for establishing an online game addiction diagnosis. However, it must be emphasized that the cut-off score provided was just an estimate. By no means can the cut-off score be used as a sole diagnostic tool for online game addiction diagnosis.

Moreover, this research may contribute by providing an estimate of online game addiction diagnosis in the Indonesian school students' population. The estimated online game addiction cases (10.15%) among Indonesian school students that currently played online games are quite alarming. In comparison, Korea estimated 2.4% game addiction cases and 10.2% ‘borderline’ cases among population aged 9 to 39 years old[25], and China estimated 13.7% of adolescent internet uses meet the internet addiction diagnostic criteria[13]. Meanwhile, data from United States and Europe showed an estimate of 1.5% to 8.2% of internet addiction cases[26]. At first sight, the estimated percentage of Indonesian online game addiction cases is comparable to other countries. But, it must be noted that the Indonesian data is highly specific to online game, whereas data from other countries have a wider scope. The data from Korea showed game addiction, which include both, online and non-online gamers. Whereas, the data from China, United States, and Europe showed internet addiction which includes an even larger range of activities, such as: chatting, browsing, and online games. Therefore, Indonesia has comparable estimated prevalence with other countries, yet it is more specific in nature. This might convey that there is an even larger undetected addiction cases under the broader category of internet addiction or game addiction.

There are limitations of the Indonesian Online Game Addiction Questionnaire. First of all, the measure only has limited clinical use. The Indonesian Online Game Addiction Questionnaire lacks data from clinical population that is established by clinical diagnosis of mental health professionals. Therefore, any conclusions drawn from the provided cut-off score can be no more than an estimate. Secondly, the Indonesian Online Game Addiction Questionnaire is constructed based on school student sample. Its use outside the context of school students warrants further investigation (i.e. adult population).

## Conclusion

The Indonesian Online Game Addiction Questionnaire has acceptable psychometric evidence for research use on online game addiction among Indonesian school students. In addition, it has a clinical cut-off to estimate the prevalence of mild addiction and addiction cases on a population. The cut-off may play a supporting role in helping mental health professionals to diagnose online game addiction. However, much work is needed before it can be used as a clinical instrument.
